# Inhibitory Effect of *Rosa rugosa* Tea Extract on the Formation of Heterocyclic Amines in Meat Patties at Different Temperatures

**DOI:** 10.3390/molecules21020173

**Published:** 2016-01-30

**Authors:** Muneer Ahmed Jamali, Yawei Zhang, Hui Teng, Shun Li, Fulong Wang, Zengqi Peng

**Affiliations:** 1 College of Food Science and Technology, National Center of Meat Quality and Safety Control, Nanjing Agricultural University, Nanjing 210095, China; majamali65@yahoo.com (M.A.J.); 2011208016@njau.edu.cn (Y.Z.); 2011208015@njau.edu.cn (H.T.); 2014108046@njau.edu.cn (S.L.); 2012208013@njau.edu.cn (F.W.); 2 Jiangsu Synergetic Innovation Center of Meat Production, Processing and Quality Control, Nanjing 210095, China

**Keywords:** *Rosa rugosa* tea, heterocyclic amines, meat patties, beef

## Abstract

In previous studies, heterocyclic amines (HCAs) have been identified as carcinogenic and a risk factor for human cancer. Therefore, the present study was designed to identify bioactive natural products capable of controlling the formation of HCAs during cooking. For this purpose we have evaluated the effect of *Rosa rugosa* tea extract (RTE) on the formation of HCAs in ground beef patties fried at 160 °C or 220 °C. RTE is rich in phenolic compounds and capable of inhibiting the formation of free radicals. The pyrido[3,4-*b*]indole (norharman) and 1-methyl-9*H*-pyrido[3,4-*b*]indole (harman) contents were significantly (*p* < 0.05) decreased in RTE-treated patties at 220 °C. 9*H*-3-Amino-1-methyl-5*H*-pyrido[4,3-*b*]indole acetate (Trp-P-2) and 3-amino-1,4-dimethyl-5*H*-pyrido-[4,3-*b*]indole acetate (Trp-P-1) were not detected at 160 °C and were statistically (*p* < 0.01) reduced at 220 °C compared to the control. RTE remarkably inhibited the formation of 2-amino-1-methyl-6-phenylimidazo[4,5-*b*]pyridine (PhIP) at 220 °C (*p* < 0.001) and at 160 °C (*p* < 0.05). 2-Amino-9*H*-pyrido[2,3-*b*]indole (AαC) and 2-amino-3-methyl-9*H*-pyrido[2,3-*b*]-indole (MeAαC) were only detected in the control group at 160 °C but were comparatively (*p* > 0.05) similar in the control and treated groups at 220 °C. 2-Amino-3-methylimidazo[4,5-*f*]quinoline (IQ), 2-amino-3,4-dimethylimidazo[4,5-*f*]quinoline (MeIQ), 2-amino-3,8-dimethylimidazo[4,5-*f*]quinoxaline (MeIQx), and 2-amino-3,4,8-trimethylimidazo[4,5-*f*]-quinoxaline (4,8-DiMeIQx) were not detected in any sample. Total HCAs were positively correlated with cooking loss. In the RTE-treated groups, 75% of the total HCAs were decreased at 160 °C and 46% at 220 °C, suggesting that RTE is effective at both temperatures and can be used during cooking at high temperatures to lessen the amount of HCAs formed.

## 1. Introduction

Meat is a highly nutritious food for human growth and development, but a number of studies show a possible association between the consumption of meat and an increased risk of cancer [1]. A reaction between creatine, sugars and amino acids occurs at high temperatures (above 150 °C) during the cooking of meat and causes the formation of heterocyclic amines (HCAs) in meat and meat products [2]. These HCAs are potent carcinogens and a risk factor for human cancer [3]. Until now, more than 25 types of HCAs have been identified [4]. HCAs are also present in incineration ash, tobacco smoke condensate and diesel-exhaust particles, but the most important source of exposure to HCAs occurs from high temperature cooked meat products [5]. Moreover, HCAs produced even during common household cooking procedures [6] due to high temperature, subsequently result in more accumulation of HCA content [7]. Basically HCAs belong to a class of structurally similar compounds having three fused aromatic rings possessing at least one nitrogen atom, one exocyclic amino group and up to four methyl groups [8]. Based on this chemical constitution, HCAs can be classified into two groups; the aminocarbolines and the aminoimidazoazaarenes. Carbolines are further subdivided into α, β and γ-carbolines. Aminocarbolines or pyrolytic HCAs are formed at higher temperatures (more than 250 °C). Harman and norharman are aminocarbolines known as “co-mutagens” because they do not show mutagenicity to *Salmonella* serovar *typhimurium* [9,10]. Another group called “thermic” HCAs are formed at temperatures between 150 °C and 250 °C [11,12]. The International Agency for Research on Cancer (IARC) has reported 2-amino-3,8-dimethylimidazo[4,5-*f*]quinoxaline (MeIQx), 2-amino-3,4-dimethylimidazo[4,5*-f*]quinoline (MeIQ), and 2-amino-1-methyl-6-phenylimidazo[4,5-*b*]pyridine (PhIP) as *possible human carcinogens* and 2-amino-3-methylimidazo[4,5-*f*]quinoline (IQ) as a *probable human carcinogen* and has recommended that exposure to these compounds be minimized [13]. Therefore, concentrations of HCAs in meat products should be minimized by applying different approaches. Antioxidants are well known for the reduction of HCAs in cooked meat. However, in the common household cooking of meat, commercial antioxidants are not very easy to apply, and synthetic antioxidants such as butylated hydroxytoluene and butylated hydroxyanisole have already been banned in many countries; consequently, natural antioxidants have been receiving much attention in this context.

Roses are known as edible and have been used in their fresh form or in processed products such as confectioneries and beverages. *Rosa rugosa* is a natural antioxidant plant commonly used in the manufacture of wines, teas, juices and jams [14]. High antioxidant activity and phenolic compound levels were observed in *Rosa rugosa* by Altıner and Kılıçgün [15]. Many polyphenolic compounds including gallic acid, catechin, epicatechin, epigallocatechin gallate, epicatechin gallate, quercetin, benzoic acid, quercetin glucoside, tannin and kaempferol were previously reported in *Rosa rugosa* tea [16], and polyphenolic compounds may reduce the formation of HCAs in cooked meat. Therefore, the present study was conceived. Recently, *Rosa rugosa* tea has attracted the attention of many researchers, who have determined its potential health benefits, which are expected to enhance the applications of *Rosa rugosa* tea in functional food products. To the best of our knowledge, the application of *Rosa rugosa* tea in meat products has not yet been reported in the literature, therefore, the objectives of the present study were to evaluate the total phenolic compounds, total antioxidant capacity and radical scavenging activity of *Rosa rugosa* tea extract and the effect of *Rosa rugosa* tea extract on the formation of HCAs and certain quality characteristics of ground beef patties fried at 160 °C and 220 °C.

## 2. Results and Discussion

### 2.1. Total Phenolic Compounds (TPC),Total Antioxidant Capacity and Radical Scavenging Activity

**Figure 1:**
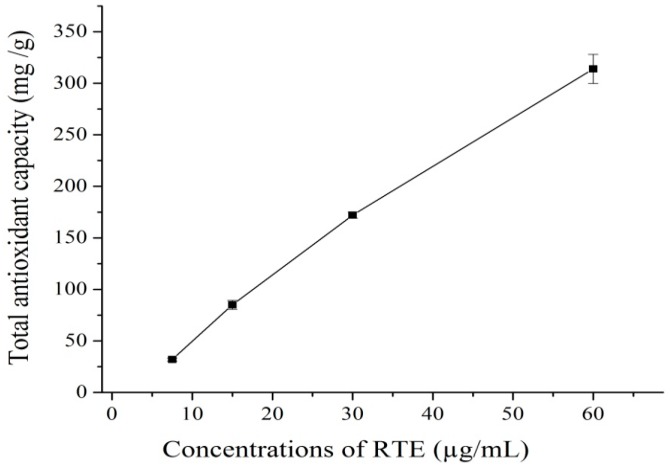
Total antioxidant capacity (mg of ascorbic acid equivalents/g dry weight) of the *Rosa rugosa* tea extract (RTE); means ± SD of triplicate analyses.

**Figure 2:**
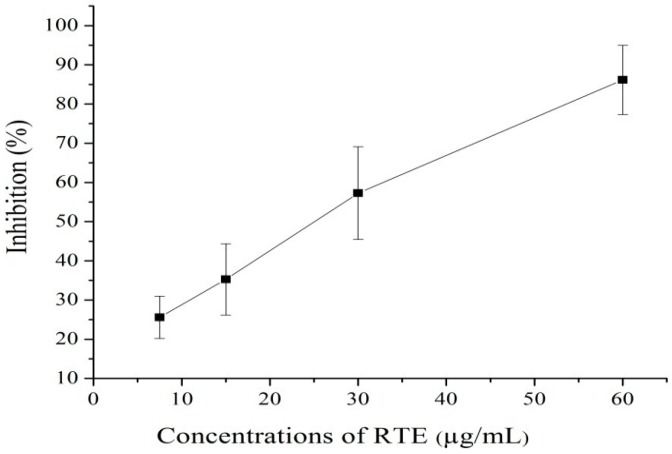
Radical scavenging activity of the *Rosa rugosa* tea extract (RTE); means ± SD of triplicate analyses.

Total phenolic compounds (TPC) act as antioxidants or free radical inhibitors. The amount of TPC in *Rosa rugosa* tea extract was 68 mg GAE/g of dry matter, and these results agree with Vinokur, *et al.*, [17], who reported a total phenolic content range in rose teas of 50.7 mg to 119.5 mg GAE/g of dry matter. Banerjee, *et al.*[18] found a similar concentration of 63 mg GAE/g dry weight in broccoli powder. The total antioxidant capacity (phosphomolybdenum method) is quantitative since the total antioxidant capacity is expressed as the number of equivalents of ascorbic acid. This assay method depends on the reduction of Mo(VI) to Mo(V) by antioxidants and the formation of a green phosphate/Mo(V) complex [19]. Figure 1 shows that the antioxidant capacity of RTE increased with increasing concentrations. Negi *et al.*, [20] and Li *et al.*, [21] reported that the antioxidant capacity of the extract shows an increasing trend with increasing concentration. Moreover, the results of the present study are similar to those reported in [19,22]. The absorbance of DPPH decreased with the addition of RTE because of radical scavenging by hydrogen donation. Figure 2 shows that the radical scavenging percentage increased with increasing concentrations of RTE. These results agree with those of Yang, *et al.*, [23]. In addition, the IC_50_ value (inhibitory concentration 50) for the inhibition of 50% of the radicals was 27.2 μg/mL.

### 2.2. Cooking loss, pH and Color

**Figure 3:**
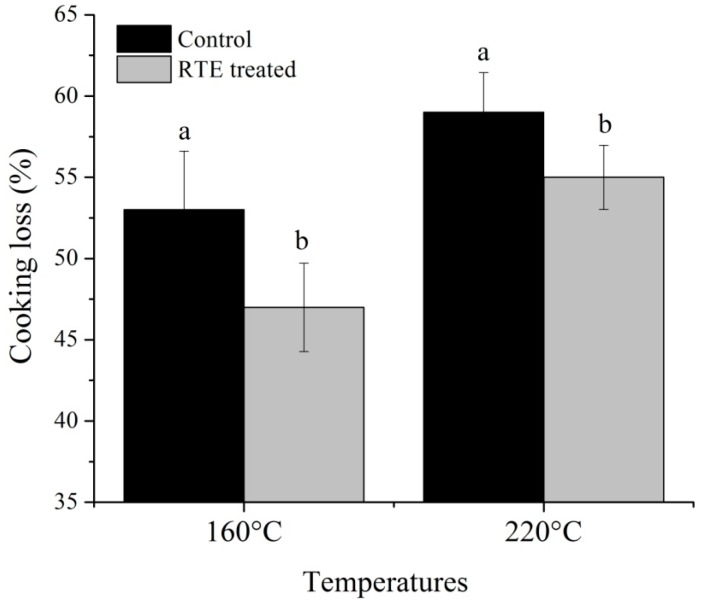
Effects of the *Rosa rugosa* tea extract (RTE) on the cooking loss; means ± SD of triplicate analyses; means bearing different letters (a, b) between control and RTE treated groups of single temperature indicate significant difference (*p* < 0.05).

**Figure 4:**
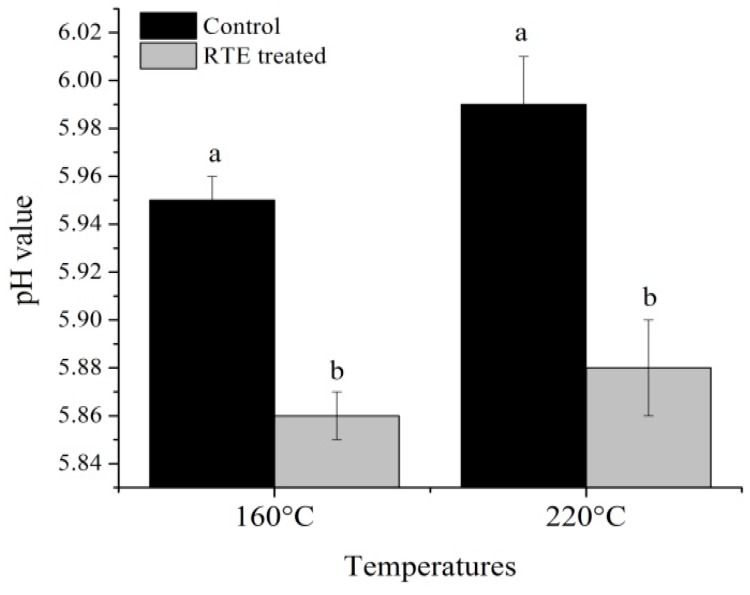
Effects of the *Rosa rugosa* tea extract (RTE) on the pH value; means ± SD of triplicate analyses; means bearing different letters (a, b) between control and RTE treated groups of single temperature indicate significant difference (*p* < 0.05).

**Figure 5:**
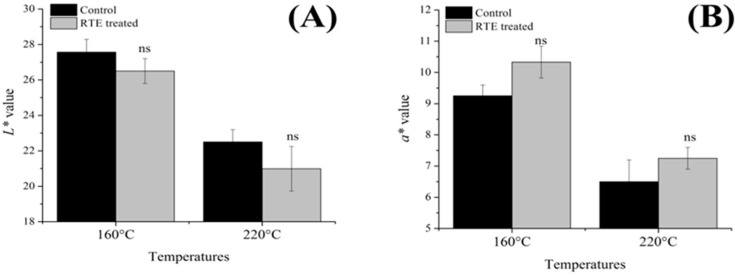

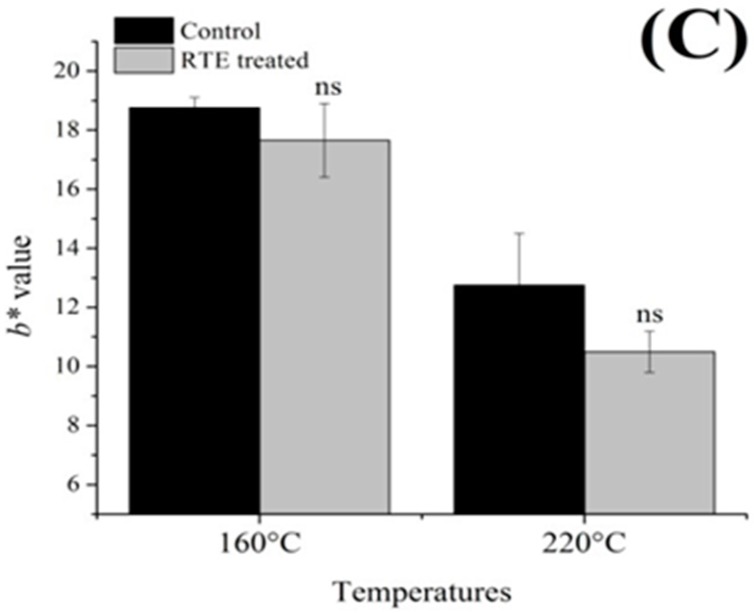
Effects of the *Rosa rugosa* tea extract (RTE) on the color value; (**A**–**C**) for L*, a*, and b*, respectively; means ± SD of triplicate analyses; ns = not significant (*p* > 0.05).

Figure 3 and Figure 4 shows the cooking loss and pH, respectively, and Figure 5 shows the color value, A, B and C for L*, a*, and b*, respectively, of patties fried at 160 °C and 220 °C with or without RTE. Cooking loss is a major factor affecting the quality characteristics of meat products. In the present study, the cooking loss ranged from 47.89% to 59.22%, and increasing temperature caused an increase in cooking loss. These results are in accordance with Oz, *et al.*, [24]. Sánchez, *et al.*, [25] concluded that the evaporation of water increased at higher temperatures and caused myofibrillar proteins to shrink, resulting in a decrease in the myofibrils’ ability to hold water. Gerber, *et al.*, [26] further reported that other compounds, such as sarcoplasmic and myofibrillar proteins, lipids, collagen, polyphosphates and salt, were all reduced along with the water content due to this increased moisture loss and subsequent increase in cooking loss of the final product. In the present study, we found that RTE significantly (*p* < 0.05) reduced the cooking loss at both temperatures; the lowest cooking losses of the antioxidant-added groups could suggest that the antioxidants have a protective role against protein denaturation. However, Keşkekoğlu and Üren [27] noted that the presence of pomegranate seed extract had no significant effect (*p* > 0.05) on the cooking loss of meatballs. Our study further demonstrated that the pH was significantly (*p >* 0.05) lower at both temperatures in the RTE-treated patties compared to the control groups, potentially due to the presence of phenolic compounds in the natural extract, which reduced the pH of the final product. Lara, *et al.*, [28] reported that the cooking loss and pH were significantly reduced by antioxidants and that the color indicators were significantly affected by natural extracts. Conversely, in our study, L*, a*, and b* were statistically (*p* > 0.05) similar, but a slight decrease in the L* value and an increase in the a* value was observed in the RTE-treated groups. Jia, *et al.*, [29] suggested that the presence of antioxidants may slow down the formation of metmyoglobin by reducing lipid oxidation and this may prevent discoloration of meat products. Moreover, all color parameters were decreased more at 220 °C compared to 160 °C, and these results agree with Oz, *et al.*, [24]. 

### 2.3. HCA Contents

**Figure 6:**
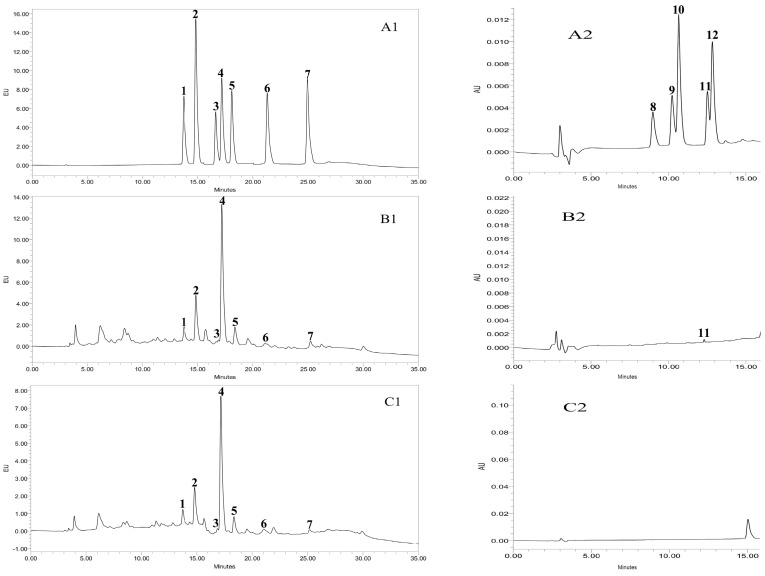
HPLC chromatograms of the HCA standard mix (**A1**) for Non-IQ-type HCAs (10 ng/mL) by fluorescence detection and (**A2**) for IQ-type HCAs (100 ng/mL) by UV detection; (**B1**,**B2**), HCAs in fried beef patties without the *Rosa rugosa* tea extract (control) at 220 °C detected by fluorescence and UV detection, respectively; (**C1**,**C2**), HCAs in fried patties with the *Rosa rugosa* tea extract at 220 °C detected by fluorescence and UV detection, respectively. Peaks: 1. Norharman, 2. Harman, 3. Trp-P-2, 4. PhIP, 5. Trp-P-1, 6. AαC, 7. MeAαC, 8. IQ, 9. MeIQ, 10. MeIQx, 11. 7,8-DiMeIQx, 12. 4,8-DiMeIQx.

The HCA content data are presented in Table 1 and HPLC chromatograms are presented in Figure 6. Norharman and harman were detected in all samples fried at 160 °C and 220 °C and the contents increased with increasing temperature. The concentration of harman in the present study agrees with the results of Gibis, *et al.*, [30], who reported that the norharman and harman contents were higher at 200–220 °C than at 150–170 °C. Several other studies have concluded that the norharman and harman contents were low in foods cooked at 150 °C, but drastically increased at temperatures higher than 190 °C [31]. The effect of the RTE on the reduction of the norharman and harman contents was different at the two different temperatures. At 160 °C, the norharman and harman contents were comparatively (*p* > 0.05) similar, whereas at 220 °C, the concentrations of norharman and harman were significantly (*p* < 0.05) reduced in the RTE-treated patties compared to the control group. These results show that the RTE was more effective against norharman and harman at 220 °C than at 160 °C, potentially due to the increased antioxidant capacity of RTE at 220 °C. Jeong, *et al.* [32] stated that the antioxidant capacity of a natural extract was significantly improved at higher temperatures. Xu, *et al.*, [33] concluded that heat treatment could be used to increase the antioxidant activity of a natural extract. Furthermore, Dong, *et al.*, [34] reported comparable concentrations of norharman and harman and noted that addition of Korean bramble and onion decreased the concentrations of norharman and harman in beef patties fried at 230 °C for 8 min. Similarly, in our study, the concentration of harman at 220 °C was 5.54 ± 0.51 ng/g in the control group and was statistically (*p* < 0.05) reduced (3.79 ± 0.02 ng/g) in the RTE-treated patties. The reduction of HCAs was due to the addition of RTE because the addition of catechins [35] and tea polyphenols [36] reduced the formation of HCAs in a model system and cooked patties.

Trp-P-2 and Trp-P-1 were not detected in any sample fried at 160 °C (Table 1) and were only detected at 220 °C because the heat treatment increased the amount and types of HCAs. Based on the available literature, Trp-P-1 and Trp-P-2 are not commonly detected HCAs [37]. However, these compounds were detected in the present study possibly because of the high fat content of the patties used; fat is known as good heat transfer agent [38]. Moreover, the concentrations of Trp-P-1 and Trp-P-2 in our study are in accordance with the results of Yao, *et al.*, [39]. In the present study, the RTE statistically (*p* < 0.01) reduced the formation of Trp-P-1 and Trp-P-2 in patties compared to the control. The inhibition might be a result of the natural extract, which decreases free radicals, as well as the pyrazine and Maillard intermediates in cooked beef. 

**Table 1:** Concentration of heterocyclic amines (ng/g) in beef patties with or without RTE fried at 160 °C and 220 °C

Temperatures		Norharman	Harman	Trp-P-2	PhIP	Trp-P-1	AαC	MeAαC	7,8-DiMeIQx	Other HCAs
160 °C	Control	0.53 ± 0.28	0.40 ± 0.14	nd	1.85 ± 0.98	nd	0.59 ± 0.21	1.10 ± 0.91	nd	nd
	RTE	0.48 ± 0.28	0.46 ± 0.02	nd	0.18 ± 0.04	nd	nd	nd	nd	nd
	Significance	ns	ns		*		-	-		
220 °C	Control	5.75 ± 0.46	5.54 ± 0.51	1.09 ± 0.42	28.05 ± 3.32	3.35 ± 0.63	0.73 ± 0.13	0.60 ± 0.14	0.52 ± 0.01	nd
	RTE	3.42 ± 0.59	3.79 ± 0.02	0.27 ± 0.05	14.0 ± 3.10	1.84 ± 0.47	0.64 ± 0.05	0.66 ± 0.40	nd	nd
	Significance	*	*	**	***	**	ns	ns	-	

RTE = *Rosa rugosa* tea extract; Control = without RTE; ns = not significant (*p* > 0.05); nd= not detected; * = significant (*p* < 0.05); ** = significant (*p* < 0.01); *** = significant (*p* < 0.001); - = Means were not compared for significance difference; Results are presented as the mean ± SD of triplicate analyses.

In the present study, PhIP was a major HCA, with more than 50% of the total HCAs containing only PhIP. A presence of PhIP was detected in all of the fried samples. The formation of PhIP was remarkably increased with increasing temperature (Table 1). This increased concentration was a result of the increased cooking loss at 220 °C. Persson, *et al.*, [40] concluded that water loss increases and HCA precursors migrate to the surface of meat at high temperatures; as a result, the formation of HCAs was increased at high temperatures. The results of the present study are similar to those of Skog, *et al.*, [41], who reported that the formation of PhIP was favored by dry conditions. Moreover, at 160 °C in our study, the amount of PhIP in the control group was 1.85 ± 0.98 ng/g and was significantly (*p* < 0.05) reduced in the RTE-treated patties (0.18 ± 0.04 ng/g). At 220 °C, the concentration of PhIP was 28.05 ± 3.32 in the control group and remarkably (*p* < 0.001) reduced to 14.0 ± 3.10 ng/g in the RTE-treated group. A similar amount of PhIP, 31.80 ng/g at 225 °C, was observed in control meatballs [42], which was reduced by the surface application of black pepper. Balogh, *et al.*, [31] found that the concentration of PhIP ranged from 0.9 to 31.4 ng/g for different time and temperature combinations. A similar amount of PhIP was reported in a control group [43], which decreased in marinated samples with green tea extract. Our results confirm that RTE was highly effective in reducing the formation of PhIP at both temperatures. Vitaglione and Fogliano [44] reported that antioxidants may reduce the formation of carcinogenic HCAs through different pathways, free radical scavenger activity or radical quenchers. 

The AαC and MeAαC contents were 0.59 ± 0.21 and 1.10 ± 0.91 ng/g, respectively, at 160 °C in the control patties and were not detected in the RTE-treated samples. Similarly, in [45], AαC was detected only in control cooked beef at 210 °C for 20 min and was not detected in 0.5% and 1.0% Herbalox extract-treated groups. However, at 220 °C in our study, AαC and MeAαC were detected in both groups (control and RTE-treated), and the statistical analysis showed that the effect of RTE was not significantly (*p* > 0.05) different in the control and treated groups. Liao, *et al.*, [46] noted similar results in which the concentrations of MeAαC were statistically similar in the control group and in groups with different levels of added vitamin C and E. In our study, the concentration of 7,8-DiMeIQx was 0.52 ± 0.01 ng/g and was only detected in the control group at 220 °C, and IQ, MeIQ, MeIQx, and 4,8-DiMeIQx were not detected in any sample. Wang, *et al.*’s data [47] supports our results; IQ, MeIQ, MeIQx and 4,8-DiMeIQx were not detected when patties were fried at 150 °C to 220 °C for 10 min. IQ was not detected at 150–250 °C [48,49]. IQ and MeIQx were not detectable in cooked offal in [50]. Warzecha, *et al.*, [6] reported that IQ was not detected in beef cooked at 150–160 °C for 20 min. In addition, IQ was detectable in beef after cooking at 225 °C for 20 min, but its amount was not quantified [51]. IQ, MeIQx and MeIQ were not detected after sous-vide cooking for 120 min [52]. Oz, *et al.*, [24] determined that 4,8-DiMeIQx was not detected between 150–250 °C and MeIQx was only detected at 250 °C. Moreover, it is well known from the literature that these compounds (IQ, MeIQ, MeIQx, and 4,8-DiMeIQx) belong to the thermic group of HCAs and form at high temperatures and long cooking times. As a result, the fact that these HCAs were not detected in our study might be due to the shorter cooking time used in our experiments.

**Figure 7:**
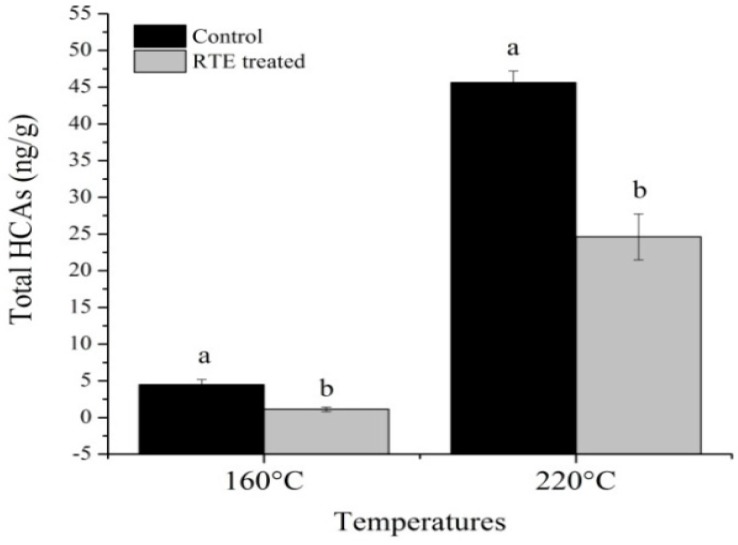
Effects of the *Rosa rugosa* tea extract (RTE) on the total HCAs; means ± SD of triplicate analyses; means bearing different letters (a, b) between control and RTE treated groups of single temperature indicate significant difference (*p* < 0.05).

As shown in Figure 7, the total HCAs were positively correlated with the cooking loss, in accordance with the literature, and increased at 220 °C compared at 160 °C. High cooking loss is related to the formation of large amounts of HCAs [53,54]. This relationship maybe due to increased water loss, decreased water binding ingredients and migration of HCA precursors to the surface of the meat. Persson, *et al.*, [55] reported that increased cooking loss was related to the increased amount of HCAs. Another study also showed that reduced cooking loss during heating reduced the transport of water and water-soluble precursors to the surface of meat products, which may cause decrease in amount of HCAs [40]. Therefore, adding water-binding ingredients may limit the transportation of precursors from the interior part of the food to the surface and cause a decrease in HCA formation [56]. For this reason reducing cooking loss is an effective approach to decrease HCA formation in meat products. In our study, the total HCAs refers to only non-polar HCAs because polar HCAs, except 7,8-DiMeIQx, were not detected in the control group at 220 °C. The total amount of HCAs in the control groups were 4.47 and 45.11 ng/g at 160 °C and 220 °C, respectively. Knize and Felton, [56] concluded that the beef patties cooked at 140 °C had low concentrations of HCAs and the amount of HCAs became higher when the temperature increased from 140 °C to 250 °C. Our results further demonstrate that the total HCAs were decreased in the RTE-treated groups compared with the control groups. In the RTE-treated groups, 75% of the total HCAs were decreased at 160 °C and 46% at 220 °C. Cai, *et al.*, [57] revealed similar results and stated that phenolic compounds from natural extracts were bioactive and played a major role in the prevention of carcinogenic compound formation. Furthermore, the radical scavenging activity and antioxidant activity of phenolic compounds are based on the methoxy substituents in the molecules and the position and number of hydroxyl groups.

Heat treatment increases the mass transfer of water containing precursors migrating from the center to the surface of the meat product, and as a result formation of HCAs in meat products is increased [58]. The difference in the reduction of HCAs at different temperatures may be due to the fact that the increase in temperature may cause changes in the molecules of antioxidant, thus change in the mechanism of initiation or propagation properties, as a results difference in effectiveness of antioxidants [59]. The use of phenolic compounds to reduce the HCAs in beef products is widely available in the literature [60]. The Maillard reaction and radicals play an important role during the formation of HCAs [11]. However, the reactive intermediates (pyrazinum and pyridinum cation radicals) can be inactivated by the antioxidative effect of antioxidants. Yoshida *et al.*, [61] reported that the formation of HCAs depends on free radical reactions and antioxidants have inhibitory effects. Kikugawa *et al.*, [62] further confirmed that influence of free radicals in the formation of HCAs and reduction by phenolic compounds. Moreover, a recent study [63] showed that the reaction between phenolic compounds and the carbonyl group of phenylacetaldehyde may generate new products. These products could disturb the reaction with creatinine and influence the aldol condensation product formation, and finally reduce the formation of PhIP. Phenolic compounds in tea, such as epigallocatechin (EGC), epicatechin gallate (ECG), epigallocatechin gallate and aflavin-3,3-digallate, possessed the highest antioxidant properties and reduced the formation of HCAs [64]. RTE comprises these compounds [16] and, as a result, can effectively reduce the formation of HCAs.

## 3. Experimental Section

### 3.1. Chemicals 

Twelve types of HCA standards including pyrido[3,4-*b*]indole (norharman), 1-methyl-9*H*-pyrido[3,4-*b*]indole (harman), 9*H*-3-amino-1-methyl-5*H*-pyrido[4,3-*b*]indole acetate (Trp-P-2), 3-amino-1,4-dimethyl-5*H-*pyrido[4,3-*b*]indole acetate (Trp-P-1), 2-amino-1-methyl-6-phenylimidazo-[4,5-*b*]pyridine (PhIP), 2-amino-9*H*-pyrido[2,3-*b*]indole (AαC), 2-amino-3-methyl-9*H*-pyrido[2,3-*b*]-indole (MeAαC), 2-amino-3-methylimidazo[4,5-*f*]quinoline (IQ), 2-amino-3,4-dimethylimidazo[4,5-*f*]quinoline (MeIQ), 2-amino-3,8-dimethylimidazo[4,5-*f*]quinoxaline (MeIQx), 2-amino-3,4,8-trimethylimidazo[4,5-*f*]quinoxaline (4,8-DiMeIQx) and 2-amino-3,7,8-trimethylimidazo[4,5-*f*]-quinoxaline (7,8-DiMeIQx) were purchased from Toronto Research Chemicals (Downsview, ON, Canada). Stock solutions of HCAs were prepared with methanol and stored in volumetric flasks at 4 °C until further dilution. Analytical-grade chemicals including sodium hydroxide, hydrochloric acid, ammonium acetate and ammonia were purchased from Sinopharm Chemical Reagent Co., Ltd. (Shanghai, China). HPLC-grade solvents including acetic acid, dichloromethane and methanol were purchased from Tedia Co. (Fairfield, OH, USA).

### 3.2. Instrumentation

Supelclean™ LC- 18 cartridges were purchased from Supelco Analytical (Bellefonte, PA, USA), and Bond Elut PRS cartridges (3 mL) were purchased from Varian Co. (Lake Forest, CA, USA). Solid-phase extraction was performed using a vacuum manifold (CNW Technologies GmbH, Düsseldorf, Germany). A reversed-phase TSK gel ODS-80 TM column (25 cm, 4.6 mm, 5 µm, 80 Å, Tosoh, Tokyo, Japan) was used for separation.

### 3.3. Rosa rugosa Tea and Preparation of the Extract

*Rosa rugosa* tea was purchased from a local supermarket in Nanjing, China, and brought to the laboratory, where it was washed under tap water. After drying at 40 °C, the tea was pulverized and vacuum-packaged and then stored at −20 °C before extraction. For extraction, 70% ethanol was prepared with distilled water (*v*/*v*). The fine powder of *Rosa rugosa* tea was mixed with ethanol (1:20 *w*/*v*) and placed on orbital shaker at room temperature. After 24 h, the extract was filtered through filter paper (Whatman no. 1) and concentrated at 50 °C using a rotary evaporator. Extra solvent was removed using a freeze dryer, and the freeze-dried extract was stored at −20 °C before use. 

### 3.4. Determination of the Total Phenolic Compounds, Total Antioxidant Capacity and Radical Scavenging Activity of Rosa Rugosa Tea Extract (RTE)

The total phenolic content of the RTE was measured by the Folin–Ciocalteu method [65]. A standard curve (r^2^ = 0.997) was plotted using different concentrations of gallic acid, and the absorbance values and results were expressed as mg of gallic acid equivalents/g of dry matter. The total antioxidant activity of the extract was carried out by the phosphomolybdenum method [66]with minor modifications as reported in our previous study [21]. A standard curve (r^2^ = 0.993) was constructed using different concentrations of ascorbic acid, and results were expressed as mg of ascorbic acid equivalents/g of dry weight. The radical scavenging activity of the RTE was determined using the DPPH method, as reported by Nowak, *et al.*, [67]. The results of the radical scavenging activity assay were expressed as the inhibition percentage of radicals using the following equation:

(1) $$\text{Radical scavenging activity }\%=\frac{Acontrol-Asample}{Acontrol}\times 100$$

where *A_control_* is the absorbance of DPPH without the RTE and *A_sample_* is the absorbance of the reaction mixture of the RTE with DPPH. The dose response curve (r^2^ = 0.989) was plotted to calculate the inhibition of 50% of the DPPH radicals (IC_50_) at different concentrations (7.5, 15, 30 and 60 μg/mL) of RTE and the percent inhibition of radicals. All determinations were performed in triplicate.

### 3.5. Preparation and Frying of Beef Patties

Meat samples were obtained from a local market and used to prepare the patties. First, the meat samples were thawed at 4 °C for 10 h and ground through a plate grinder. The fat content was adjusted to 25%, and 0.1% of *Rosa rugosa* tea extract (RTE) was added. After mixing, the uniformly ground meat was shaped into patties. The weight of each patty was 50 g, and the size of each one was approximately 5 cm in diameter and 2.5 cm thick. Control samples were prepared the same as above but without the addition of the RTE. After 12 h, the patties were subjected to deep fat frying in a commercial stainless steel deep-fat fryer. Refined soybean oil was used for frying because it is the most commonly used oil in China. The temperature was carefully monitored by an infrared thermometer; when the temperature of the oil reached a specific point, the patties were deep fat fried at two different temperatures, *i.e.*, 160 °C and 220 °C ± 2 for 7 min. After frying, once the temperature of the samples had decreased to room temperature, the cooked weight and color of the patties were analyzed, and the patties were then ground to obtain uniform samples using a kitchen mixer and stored at −20 °C until further analysis. Samples were thawed at 4 °C for approximately 16 h prior to analysis. All experiments were repeated three times, and all determinations were performed in triplicate at room temperature. 

### 3.6. Determination of Cooking Loss, pH and Color

The cooking loss during cooking was measured as the weight difference between raw and cooked patties. For the determination of pH, 10 g of each patty was homogenized with 50 mL of distilled water, and the pH was measured using a digital pH meter, which was calibrated using a buffer solution. The color of the patties was measured as L*, a*, and b* for lightness, redness and yellowness, respectively, using a colorimeter (Konica Minolta Co., Tokyo, Japan). The colorimeter was calibrated with the standard white tile provided with the instrument. Triplicate measurements were performed for the analysis.

### 3.7. Extraction, Purification and Quantification of HCAs

The extraction, purification and quantification of HCAs were determined by the method reported in our previous study [68]. A high-performance liquid chromatography (HPLC) system was used. A fluorescence detector was used for non-IQ-type HCAs, and an UV detector was used for IQ-type HCAs. For the recovery rate determination, 2 g of raw sample was spiked with different concentrations of the HCA standards: 20, 100 and 500 ng/g for polar HCAs and 2, 10 and 50 ng/g for non-polar HCAs. The recovery percentages for different HCAs ranged from 68 to 98%. The retention times of the analyte peaks in the standard HCA solutions were used for identification of the HCA peaks in the samples. The peaks were further confirmed through comparison with the peaks of the HCA standard spiked samples. Calculations of the amount of HCAs in the samples were performed using a standard curve equation. The concentrations ranged from 0.1–10 ng/mL for norharman, harman, Trp-P-2, Trp-P-1, PhIP, AαC and MeAαC and 10–1000 ng/mL for IQ, MeIQ, MeIQx, 7,8-DiMeIQx and 4,8-DiMeIQx in the linearity study. The correlation coefficients for the different HCAs were 0.9992 to 0.9997. The limits of detection were 0.04, 0.02, 0.05, 0.02, 0.03, 0.03, 0.01, 2.61, 2.89, 0.61, 0.16 and 0.49 ng/g and limits of quantification 0.14, 0.07, 0.17, 0.08, 0.11, 0.12, 0.04, 7.92, 8.81, 1.92, 0.49 and 1.51 ng/g for norharman, harman, Trp-P-2, Trp-P-1, PhIP, AαC, MeAαC, IQ, MeIQ, MeIQx, 7,8-DiMeIQx and 4,8-DiMeIQx, respectively.

### 3.8. Statistical Analysis

The collected data of the present study was tabulated and analyzed for the means ± SD. The means were statistically analyzed by *t*-test for the comparison of control and RTE groups. The means were further computed for the one-way ANOVA and least significant difference (LSD) for significance levels of 0.05, 0.01 and 0.001 using SAS version 9.1 (SAS Institute Inc., Cary, NC, USA).

## 4. Conclusions 

*Rosa rugosa* tea extract is rich in phenolic compounds and capable of inhibiting free radicals. HCAs were positively correlated with cooking loss due to the increased water loss, decreased water binding ingredients and migration of HCA precursors to the surface of the meat. The amount of HCAs was increased at 220 °C compared to 160 °C. Norharman, harman and PhIP contents were detected in all ground beef samples fried at 160 °C and 220 °C. *Rosa rugosa* tea extract reduced the cooking loss and the formation of total and individual HCA and was highly effective against the formation of PhIP. In general, *Rosa rugosa* tea extract was greatly effective at both temperatures (160 °C and 220 °C) against carcinogenic HCAs. These results suggest that the temperature should be controlled and that *Rosa rugosa* tea extract can be used during cooking to decrease HCAs. The facts from the present report provide information on fried meat products and could be used to minimize the formation of HCAs. This method can be adopted and would be practical in the cooking of meat because *Rosa rugosa* tea is widely available and commonly consumed worldwide.
